# Defining the Earliest Transcriptional Steps of Chondrogenic Progenitor Specification during the Formation of the Digits in the Embryonic Limb

**DOI:** 10.1371/journal.pone.0024546

**Published:** 2011-09-13

**Authors:** Carlos I. Lorda-Diez, Juan A. Montero, Manuel J. Diaz-Mendoza, Juan A. Garcia-Porrero, Juan M. Hurle

**Affiliations:** Departamento de Anatomía y Biología Celular and Instituto de Formación e Investigación Marqués de Valdecilla, Universidad de Cantabria, Santander, Spain; Childrens Hospital Los Angeles, United States of America

## Abstract

The characterization of genes involved in the formation of cartilage is of key importance to improve cell-based cartilage regenerative therapies. Here, we have developed a suitable experimental model to identify precocious chondrogenic events *in vivo* by inducing an ectopic digit in the developing embryo. In this model, only 12 hr after the implantation of a Tgfβ bead, in the absence of increased cell proliferation, cartilage forms in undifferentiated interdigital mesoderm and in the course of development, becomes a structurally and morphologically normal digit. Systematic quantitative PCR expression analysis, together with other experimental approaches allowed us to establish 3 successive periods preceding the formation of cartilage. The “pre-condensation stage”, occurring within the first 3 hr of treatment, is characterized by the activation of connective tissue identity transcriptional factors (such as Sox9 and Scleraxis) and secreted factors (such as Activin A and the matricellular proteins CCN-1 and CCN-2) and the downregulation of the galectin CG-8. Next, the “condensation stage” is characterized by intense activation of Smad 1/5/8 BMP-signaling and increased expression of extracellular matrix components. During this period, the CCN matricellular proteins promote the expression of extracellular matrix and cell adhesion components. The third period, designated the “pre-cartilage period”, precedes the formation of molecularly identifiable cartilage by 2–3 hr and is characterized by the intensification of *Sox 9* gene expression, along with the stimulation of other pro-chondrogenic transcription factors, such as *HifIa*. In summary, this work establishes a temporal hierarchy in the regulation of pro-chondrogenic genes preceding cartilage differentiation and provides new insights into the relative roles of secreted factors and cytoskeletal regulators that direct the first steps of this process *in vivo*.

## Introduction

Chondrogenesis is an essential process in vertebrates that is responsible for the establishment of the skeletal primordia and for the subsequent growth of long bones. Abnormal cartilage development causes a large proportion of birth defects in humans. Hence, the identification and functional characterization of all the genes involved in the formation of cartilage is of key importance to improve preventive and therapeutic strategies for congenital skeletal diseases (see [1]). Additionally, detailed knowledge about the molecular regulation of chondrogenic differentiation is of critical importance for the development of efficient cell-based regenerative therapies to repair cartilage defects.

Basically, the process of chondrogenesis is initiated by the formation of a prechondrogenic blastema of condensed progenitors followed by differentiation into chondrocytes, accompanied by the production of a cartilage matrix [2]. Subsequent during development, chondrocytes may undergo hypertrophic maturation to eventually be replaced by bone via endochondral ossification. In the last several decades, the use of powerful experimental approaches, such as mouse genetics and microarray analyses, have allowed the identification of many different genes implicated in chondrogenesis, including growth/differentiation factors, extracellular matrix (ECM) genes, transcription factors and miRNAs [3]–[8]. However, we are still far from understanding the mechanisms responsible for chondrogenesis. Whether the packaging of cell progenitors into precondensation aggregates is caused by increased cell–cell contacts, increased proliferation, migration of progenitors to a central region or by a combination of these different processes is not yet clear [2]. Similarly, we do not know whether different chondrogenic systems, such as appendicular and axial skeleton in the embryo, or different in vitro stem cell lineages require the same signals for differentiation into cartilage. An additional hurdle regarding our understading of chondrogenesis is related to the absence of phenotypic stability of mature cartilage and the genetic similarities that account for the formation of cartilage and other varieties of connective tissues, primarily tendon and dense fibrous connective tissues [8]. This is particularly relevant in regenerative medicine because chondrocytes lose their phenotype in the course of amplification in culture and acquire a fibroblastoid morphology [9].

One of the reasons for our relatively limited knowledge regarding the chondrogenic process is that current experimental approaches are based on cell culture assays which cannot provide precise information about the temporal sequence of gene activation/inactivation that is responsible for chondrogenesis in vivo. Hence, differentiation in culture is not fully synchronized, and cellular aggregates at different stages of differentiation or that have differentiated following other connective tissue pathways (see [10]) exist concurrently in current culture models for chondrogenesis. Similarly, functional redundancy often makes identification of the precise involvement of a particular gene in the formation of cartilage difficult [11]–[13]. These and other reasons make it necessary to design new experimental systems to unravel the cellular and molecular mechanisms responsible for chondrogenesis.

In previous studies, we have identified Tgf-βs and Activins as triggering signals for the formation of digits in the developing limb autopod [14], [15]. On the basis of those studies, we have established a robust in vivo model for the induction of an ectopic digit in the chick embryo by local implantation of a microbead bearing Tgf-βs or Activins into the interdigital mesenchyme prior to its physiological regression via cell death [16]. The advantage of this model for the analysis of chondrogenesis is derived from the in vivo nature of the method, from the efficiency of the treatment (discarding experiments in which the bead is lost, the formation of cartilage is recognized in almost 100% of the experiments), and from the facility of establishing a time-lapse period of differentiation in hours (the most precocious regulation of chondrogenic genes was recognized half an hour after the experimental manipulation). Hence, the simplicity of the system makes it a particularly useful tool for defining the cell and molecular events occurring during in vivo chondrogenesis in detail.

In the present study, we have employed the induction of interdigital ectopic digits to characterize the sequence of gene regulation events that occur *in vivo* and to explore the functional significance of some of the most precocious factors regulated during the induction of chondrogenesis. This study provides a comprehensive time-lapse sequence analysis of gene expression during *in vivo* chondrogenesis, which allows the establishment of three sequential steps in the commitment of mesodermal progenitors towards cartilage. We further show the importance of cell motility versus cell proliferation in the formation of prechondrogenic blastemas and provide new data indicating CCN matricellular proteins as modulators of extracellular matrix production and cell-matrix adhesion of both cartilage and tendons.

## Results

### Characterization of the Experimental Model for *In Vivo* Chondrogenesis

**Figure 1:**
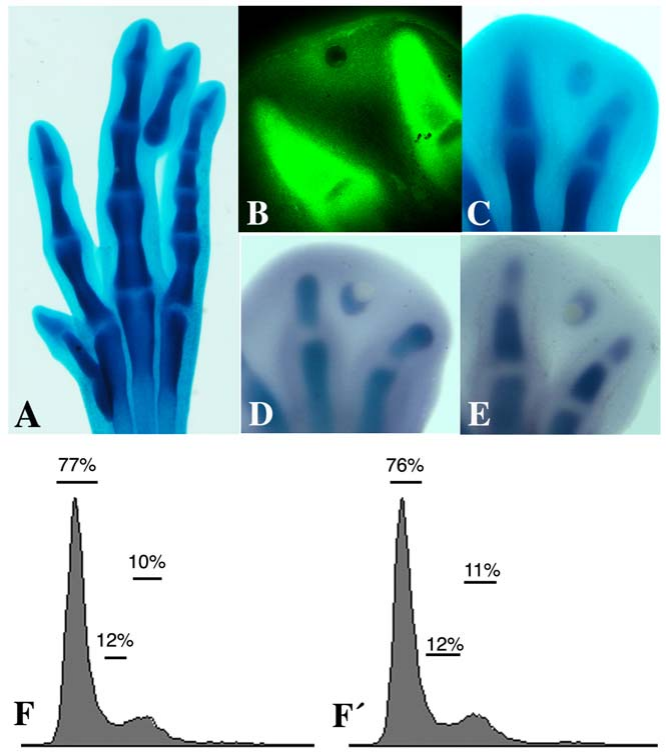
Characterization of chondrogenesis induced by interdigital application of a heparin bead incubated in 2 µg/ml Tgfβ 1. A, Morphological appearance of the extra digit 3 days after the interdigital implantation of a Tgfβ bead. B, PNA positive labeling of the interdigital mesoderm 10 hr after the implantation of a Tgfβ bead. C, Alcian blue-positive cartilage 22 hr after the implantation of a Tgfβ bead. D, presence of an ectopic aggrecan gene expression domain 12 hr after the implantation of a Tgfβ bead. E, Presence of an ectopic *collagen 2 alpha 1* expression domain 17 hr after the implantation of a Tgfβ bead. (*) Tgfβ bead; (D3) digit 3; (D4) digit4. F-F′ charts representing cell proliferation of control interdigits (F) and of those treated with a Tgf-β bead (F′) as measured by flow cytometry. The percentages of cells in the G0/G1, S, and G2/M phases of the cell cycle are shown.

Interdigital implantation of a Tgfβ bead at 5.5 id was followed by the formation of an ectopic digit that was detectable two days later by alcian blue staining in 35 out of 40 experimental embryos (87.5%; Fig. 1A). To relate the pattern of gene expression to different skeletogenic events, the time course of digit morphogenesis was monitored using peanut agglutinin labeling (PNA) as a marker of the prechondrogenic aggregate and, alcian blue staining and expression of the *type II Collagen* and *Aggrecan* genes as markers of cartilage differentiation.

Initial indication of a PNA positive prechondrogenic aggregate was observed in the interdigit 10 hr “after bead implantation” (ABI; Fig. 1B). Alcian blue staining provided a tenuous indication of the formation of ectopic cartilage between 15 and 18 hr ABI, but overt chondrification in the interdigit was not detectable by this procedure until 20 hr ABI or later (Fig. 1C). However, the presence of an ectopic domain of *Aggrecan* gene expression, which is indicative of the transition from the stage of prechondrogenic condensation to the period of cartilage differentiation, was clearly detectable 12 hr ABI (Fig. 1D). A comparable ectopic domain of *type II Collagen* gene expression also preceded the identification of cartilage by alcian blue staining by hours (Fig. 1E). Hence, the 10-hr period selected for the present study covered the stage of prechondrogenic aggregation and the commitment of the undifferentiated mesoderm to the chondrocytic lineage.

Analysis of interdigital cell proliferation using flow cytometry showed that the formation of the extradigit was not accompanied by changes in cell proliferation at 3, 6 and 10 hr ABI in comparison with the interdigit of the contralateral control limb (Fig. 1 F-F′).

### Adhesion, Cell Shape and Cell Motility Genes (Table 1)

**Table 1:** Regulation of genes involved in the control of cell adhesion at 1, 3, 6, and 10 hr after interdigital implantation of beads bearing Tgf-β1.

		Fold changes vs control			
Gene	GenBank	1 hr.	3 hr.	6 hr.	10 hr.
**Cell Adhesion**					
*N-cadherin*	NM_001001615	1.20±0.08	0.82±0.09	1.17±0.08	1.44±0.28
*Cadherin 4*	NM_001004391	1.04±0.09	1.09±0.10	0.84±0.05	1.16±0.06
*Cadherin 11*	NM_001004371	1.01±0.08	1.02±0.02	1.21±0.09	1.27±0.20
*Cadherin 13*	NM_001001760	1.15±0.51	1.26±0.07	1.11±0.09	1.38±0.30
*Cadherin 7*	NM_204187	1.23±0.22	1.16±0.22	1.65±0.37	0.85±0.12
*ITGA4*	XM_421974	0.83±0.08	1.24±0.02	0.99±0.31	1.57±0.12
*ITGAV*	NM_205439	1.04±0.01	0.73±0.06	0.89±0.07	0.93±0.25
*ITGβ1*	NM_001039254	0.92±0.14	0.92±0.10	1.27±0.17	1.14±0.21
*ITGβ3*	NM_204315	1.15±0.18	1.37±0.18	1.27±0.02	1.26±0.11
*ITGA1*	NM_205069	0.64±0.08	0.75±0.00	1.21±0.14	1.23±0.11
*ITGA5*	AY029523	0.92±0.12	1.86±0.26*	1.52±0.16*	1.98±0.37*
*ITGβ5*	NM_204483	1.12±0.02	1.20±0.18	1.43±0.22	1.57±0.34
*NCAM*	XM_001234121	0.78±0.15	1.50±0.16*	0.94±0.02	0.82±0.09
**Cytoskeletal regulators**					
β *Catenin*	NM_205081	0.82±0.05	1.17±0.03	0.77±0.09	0.90±0.04
*RhoA*	NM_204704	0.97±0.09	0.88±0.04	1.15±0.15	0.90±0.01
*RhoC*	NM_001029849	1.44±0.27	1.54±0.16*	1.64±0.16*	1.82±0.16*
**Ephrin signalling**					
*Ephrin-A2*	NM_204983	1.22±0.05	1.38±0.10	1.05±0.21	1.45±0.08
*EPH receptor A4*	NM_204781	0.84±0.09	1.32±0.22	0.99±0.06	0.99±0.14
*Ephrin-A5*	NM_205184	2.11±0.11*	0.92±0.02	0.86±0.01	1.76±0.52
*EPH receptor A7*	NM_205083	1.32±0.22	0.60±0.02	0.91±0.06	1.53±0.41
**Galectins**					
*CG-8*	NM_001010843	0.56±0.19*	0.43±0.07*	0.31±0.04**	0.65±0.05
*CG-1A*	NM_206905	0.69±0.07	1.15±0.21	1.28±0.22	1.00±0.12
*CG-1B*	NM_205495	0.91±0.01	1.26±0.06	1.39±0.42	1.98±0.04*

(*) p-value≤0.05 or (**) p-value≤0.01 or (***) p-value≤0.001 using the expression level of the gene in the contralateral untreated interdigit as the calibrator.

Many different studies have emphasized the potential importance of different adhesion molecules and cytoskeletal regulators in chondrogenic differentiation [4], [12], [17]–[20]. However, their precise role in the chondrogenic signaling cascade is obscure and mice with targeted deficiencies of the most relevant adhesion molecules show a normal pattern of appendicular skeleton [11], [13], [21].

#### Cell Adhesion

In the present study we have selected a cohort of cell adhesion molecules (Table 1) previously proposed to be involved in chondrogenesis, including cadherins [3], [11], [17], [18], [22], integrins [19] and NCAM [23], [24]).

We detected only a moderate upregulation of the *NCAM* and *alpha 5 Integrin* genes at 3 hr ABI. The upregulation of *NCAM* was transitory, but the upregulation of *alpha 5 Integrin* was maintained in subsequent stages. As shown in Table 1, other adhesion molecules appeared to be moderately upregulated at 6 and 10 hr ABI, but this upregulation did not reach statistically significant levels.

#### Cytoskeletal Modulators

**Figure 2:**
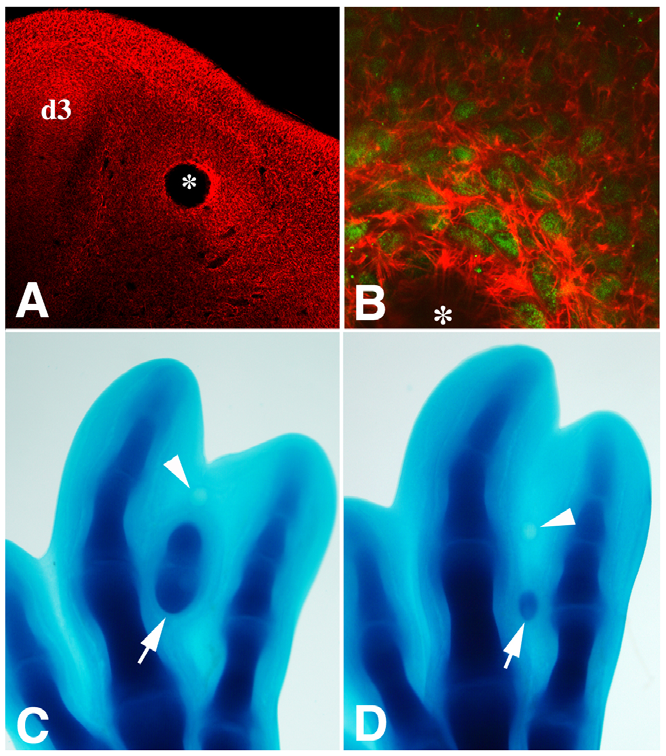
Actin cytoskeleton in digit chondrogenic induction. A, Low magnification view of a phalloidin-stained section of the autopod at 3 hr ABI to show increased staining around the bead (*) and at the digit tip of neighboring digit 3 (d3). B, Detailed view of the actin cytoskeleton of p-Smad2-positive (green nuclear labeling) cells located around the Tgfβ bead (*) 3 hr ABI. Note the increased protrusive actin-based filaments in the p-Smad2-positive cells. Digit 3 (d3), digit4 (d4). C–D, Morphology of the ectopic cartilage (arrows), 2 days after combined treatment with a Tgfβ bead and a Cytochalasin D bead (experimental, D) or a DMSO bead (control, C). Cytochalasin D (D) and DMSO (C) beads are indicated by arrowheads.

The unexpected absence of notable changes in the expression of genes responsible for increasing cell adhesion prompted us to examine cytoskeletal changes in the cells induced to form cartilage and to explore the regulation of intracellular components associated with cell adhesion and cell motility. Intracellular adhesion regulators associated with cadherins, such as β*-catenin* gene, were not regulated during the first 10 hr ABI. However, the cytoskeletal modulator *RhoC* (but not *RhoA*), which has been implicated in cell migration [25] and chondrogenesis [26], exhibited progressive upregulation at 1, 3, 6, and 10 hr ABI. In view of these findings, we examined the characteristics of the actin cytoskeleton in the treated interdigits after phalloidin labeling. As shown in Fig. 2A, 2B, at 3 hr ABI, the cells surrounding the bead exhibited a dramatic intensification and reorganization of F-actin microfilaments, which was indicative of intense mesenchymal cell remodeling in the treated interdigit. To asses the importance of these changes in the actin cytoskeleton, combined treatments were performed using Tgfβ beads and Cytochalasin D beads or, in the case of the control embryos, using a Tgfβ bead and a DMSO bead. Under these conditions, the incidence of ectopic chondrogenesis was not modified in the control embryos (10 out of 13 treated embryos; 77%), but it decreased to 43.5% (17 out of 39 cases) in the experimental embryos. Additionally, as shown in Fig. 2C, 2D, the size of the ectopic cartilage was quite reduced in most of the experimental limbs (12 out of 17). The number of dead cells induced by this treatment was very low (not shown), discarding a role for cell death in the inhibition of chondrogenesis.

#### Eph/ephrin Signaling

The ephrin family of cell surface ligands and their Eph receptor tyrosine kinases were studied because theses proteins are known to function as regulators of cell adhesion and cell–cell repulsive movements (reviewed in [27]. We have analyzed the regulation of *Ephrin A2* and *A5* genes and the genes for the receptors *Eph A4* and *A7*. Only *Ephrin A5* showed moderate upregulation at 1 hr ABI. This finding suggests an initial role of Ephrin A5 in conferring the interdigital mesenchyme with surface properties required for the formation of cartilage, consistent with the previously proposed role for ephrins in the developing autopod as downstream effectors of HoxA13 [28], [29].

#### Lectins

The regulation of endogenous lectins in chondrogenesis was explored due to their demonstrated role in mediating cell-cell and cell-matrix interactions and eliciting biosignaling [30] and because of their proposed function in cartilage differentiation [30]–[34]. Three representative members of the avian galectin gene family were selected: *CG-1A*, *CG-1B* and *CG-8*,. Beginning in the first hour ABI, *CG-8*, a galectin proposed to exert a potent negative influence in chondrogenesis [34], was downregulated. *CG-1A* and *CG-1B* exhibited no change in expression, except for a moderate upregulation of *CG-1B* observed by 10 hr ABI. However, basal expression of CG-1A and CG-1B in control interdigits was 17 times higher than that of CG-1B (not shown).

### Transcription Factors (Table 2)

**Table 2:** Regulation of transcription factor encoding genes at 1, 3, 6, and 10 hr after interdigital implantation of beads bearing Tgf-β1.

		Fold changes vs control			
Gene	GenBank	1 hr.	3 hr.	6 hr.	10 hr.
**Transcription Factors**					
*Sox9*	NM_204281	1.97±0.35*	2.30±0.12***	1.71±0.15**	9.17±3.43*
*Sox8*	NM_204731	1.07±0.20	1.08±0.19	1.89±0.44	1.35±0.02
*Scleraxis*	NM_204253	2.29±0.36*	1.78±0.33*	1.17±0.20	4.46±0.52*
*CFKH-1*	NM_205006	1.32±0.12	1.19±0.06	1.42±0.14	1.87±0.28*
*PRRX1*	NM_001007821	1.50±0.05*	1.20±0.01	1.14±0.05	1.05±0.14
*PRRX2*	XM_415476	1.20±0.15	1.45±0.23	1.04±0.11	0.85±0.41
*GATA5*	NM_205421	1.77±0.54	2.43±0.49*	1.61±0.20*	2.33±0.99
*GATA6*	NM_205420	0.91±0.01	1.35±0.15	0.88±0.10	0.74±0.13
*NKX3.2 (Bapx1)*	AF179482	0.81±0.01	0.77±0.26	0.75±0.09	0.87±0.28
*Runx2*	NM_204128	0.83±0.17	1.12±0.02	0.70±0.05	1.38±0.01
*BARX1*	NM_204193	2.43±0.49*	2.62±0.71*	1.84±0.12**	1.91±0.22*
*Pax1*	GGU22046	0.68±0.25	1.84±0.35*	1.77±0.26*	0.97±0.01
*Pax9*	NM_204912	0.72±0.08	0.83±0.12	1.19±0.45	0.78±0.10
*SnoN (SKIL)*	NM_205174	1.42±0.20	2.45±0.28**	1.11±0.09	3.65±0.38*
*Tgif1*	NM_205379	1.12±0.06	1.11±0.08	1.13±0.09	2.88±0.06**
*Hif1α*	NM_204297	1.00±0.07	1.09±0.09	0.77±0.12	1.86±0.11*

(*) p-value≤0.05 or (**) p-value≤0.01 or (***) p-value≤0.001 using the expression level of the gene in the contralateral untreated interdigit as the calibrator.

Transcription factors are key regulators of chondrogenic differentiation that may provide cartilage identity to undifferentiated mesoderm progenitors [8]. Firstly we analyzed the expression of *Sox9* as a master gene for chondrogenesis (see review [1]). We detected two expression peaks in the course of ectopic chondrogenesis. The first peak was moderate in intensity (two-fold) and was detected as early as the first hour ABI and maintained at 3 and 6 hr ABI. The second peak was detected at 10 hr ABI and was characterized by a dramatic intensification of gene expression, exhibiting up to a 14-fold increase in expression in some of the experimental samples. However, *Scleraxis*, which is considered a master gene for tendon and fibrous connective tissue formation [35], [36] also showed a transitory 2-fold increase in expression at 1 and 3 hr ABI, followed by a more intense upregulation at 10 hr ABI.

As shown in Table 2, other transcription factors with proposed roles in chondrogenesis were also regulated, including the following genes: *Gata 5*, a transcription factor proposed to modulate Tgf-β signaling during limb development [37]; *Hypoxia-inducible factor-1 alpha* (*Hif1a*; [38], [39]; the helix/forkhead domain transcription factor *cFKH 1* [40]; the homeobox-containing transcription factor *Barx1* [41], [42]; the DNA-binding paired domain gene *Pax 1* [43]; the paired-related homeobox transcription factor *PRRX1* [44], [45]; and the transcriptional co-repressors of Tgfβ signaling *SnoN* [46] and *Tgif1* [47].

Other transcription factors implicated in different chondrogenic systems were not found to be regulated in our system. These factors included *PRRX2*, *Pax9*, *Bapx1*/*Nkx3.2* [48], [49], *Runx 2* [10], *Sox8* [16], and *Gata 6* [50].

### Extracellular Matrix and Matrix Metalloproteinases (Table 3)

**Table 3:** Regulation of extracellular matrix genes at 1, 3, 6, and 10 hr after interdigital implantation of beads bearing Tgf-β1.

		Fold changes vs control			
Gene	GenBank	1 hr.	3 hr.	6 hr.	10 hr.
**Extracellular Matrix**					
*Tenascin C*	NM_205456	1.31±0.17	4.54±0.63**	1.83±0.28*	2.95±0.50*
*Versican*	NM_204787	0.73±0.07	1.02±0.26	0.81±0.10	1.12±0.07
*Decorin*	NM_001030747	0.82±0.10	1.40±0.32	0.85±0.09	0.95±0.04
*Tenomodulin*	NM_206985	1.13±0.30	1.07±0.23	0.80±0.09	1.45±0.27
*Glypican 3*	XM_001232891	1.04±0.10	1.18±0.17	0.87±0.01	1.40±0.33
*Fibronectin1*	NM_001198712	0.95±0.01	1.35±0.02	0.95±0.13	1.57±0.23*
*Ltbp1*	XM_419510	1.20±0.09	0.97±0.00	2.15±0.07**	1.76±0.01**
*Big-h3*	NM_205036	1.27±0.47	1.81±0.05***	4.06±0.02***	7.25±0.98***
**Metalloproteases**					
*Tolloid 1*	NM_204703	1.09±0.15	1.16±0.10	1.15±0.10	1.50±0.10**
*MT3-MMP*	NM_205197	0.99±0.11	1.08±0.05	1.00±0.33	1.28±0.06
**Matricellular Proteins**					
*Ccn1*	NM_001031563	2.80±0.28***	2.44±0.16***	1.31±0.10	1.66±0.20*
*Ccn2*	NM_204274	2.02±0.21**	2.14±0.28**	1.18±0.11	1.14±0.17
*Ccn3*	NM_205268	1.42±0.22	3.48±0.70*	2.93±0.18**	2.03±0.34*
*Ccn4*	NM_001024579	1.23±0.27	0.99±0.05	1.00±0.02	0.96±0.14
*Ccn5*	XM_417370	1.02±0.11	1.34±0.25	1.40±0.15	1.48±0.60
*Ccn6*	XM_001234149	0.98±0.30	0.98±0.28	0.65±0.09	0.83±0.24

(*) p-value≤0.05 or (**) p-value≤0.01 or (***) p-value≤0.001 using the expression level of the gene in the contralateral untreated interdigit as the calibrator.

The extracellular matrix constitutes a functional scaffold for differentiating chondrocytes, providing the cartilage with biomechanical properties and modulating the activity of secreted growth factors and signaling molecules. Here, we selected a sample of representative matrix components that we expected might precede the activation of specific cartilage matrix markers, such as the *type II Collagen* and *Aggrecan* genes that are not regulated up to 12 hr ABI (see Fig. 1). We observed that *Tenascin C*, was the most precocious matrix component upregulated in our experimental model, exhibiting intense increased expression at 3 hr ABI and being maintained at more moderate levels at 6 and 10 hr ABI. *Fibronectin 1* [51] was upregulated at 10 hr ABI. The remaining structural matrix components analyzed here, including *Versican*, *Decorin*, *Tenomodulin* and *Glypican 3* (see [52]–[54]), were not regulated at significant levels during the first 10 hr ABI. In contrast, *Big-h3* which is a component of some matrices with a proposed function in early stages of chondrogenesis promoting cell adhesion [55] appeared upregulated from 3 hr ABI achieving highest expression levels at 10 hr ABI. Significant upregulation was also appreciate from 6 hr ABI for *Ltbp1*, which plays a key role in the delivery of active Tgfβs in the extracellular matrix [56].

Matrix remodeling by metalloproteinases accompanied by delivery of sequestered growth factors is also an important mechanism in the regulation of cell differentiation. Many different metalloproteinases have been shown to play important functions in chondrogenesis [19], [57]. In the present study we selected two metalloproteinases, *Tolloid 1* [58] and the membrane-bound *MT3-MMP* [59], because we observed that they are highly expressed in the developing digit blastemas (not shown). However, no expression changes were appreciated in the period covered by this study except for a mild upregulation of *Tolloid 1* at 10 hr ABI.

### CCN Family of Secreted Factors (Table 3)

The CCN family of matricellular proteins contains 6 members, CCN 1–6, with functions in chondrogenesis that are still poorly characterized. Here, we observed significant and very precocious regulation of a variety of these factors, which prompted us to study their implications in our model of induced chondrogenesis in some detail.

The *Ccn1* and *Ccn2* genes were significantly upregulated in the interdigital mesenchyme following the application of Tgfβ beads. This upregulation was transitory, being detectable at 1 and 3 hr ABI. In the next period, upregulation of *Ccn1* was moderate and the expression of *Ccn2* returned to basal levels. *Ccn3* was upregulated at later time points (3 hr ABI), but its upregulation was maintained throughout the entire 10 hr period covered in this study. In contrast, *Ccn4*, *Ccn5*, and *Ccn6* were not significantly regulated during the investigated period.

**Figure 3:**
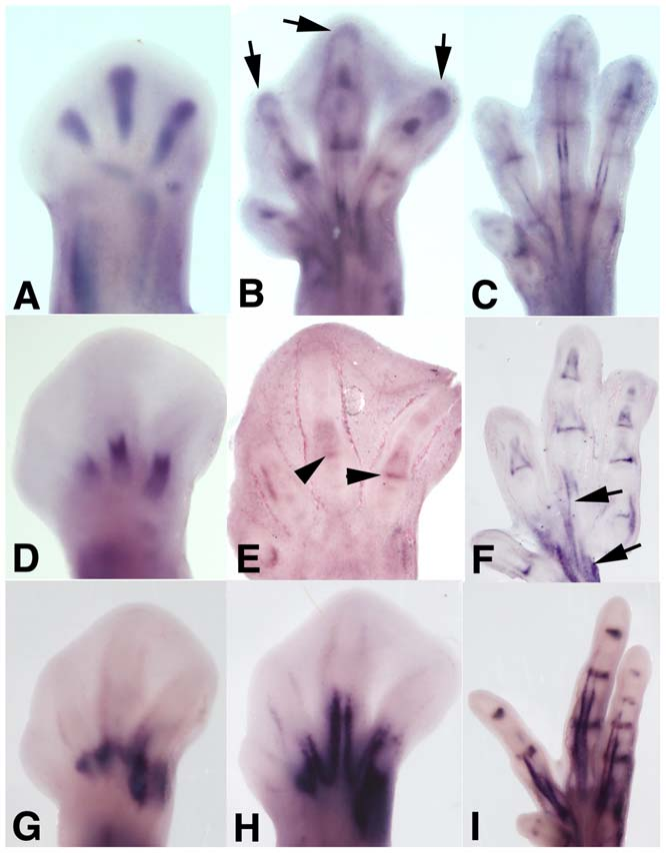
Expression of *Ccn1* (A–C), *Ccn2* (D–F), and *Ccn3* (G–I), in the developing digits at days 5,5–6,5 (A,D,G), 7–8 (B,E,H) and 8,5–9 (C, F, I) of incubation. A–C, Intense expression of *Ccn1* is first seen in the digit blastemas (A), and in the course of development, expression becomes restricted to the digit tips (arrow), joints and tendons (B). Note in C the disappearance of the digit tip domains once all of the phalanxes have formed. D–F, Expression of *Ccn2* is first associated with the zones of hypertrophic differentiation of digit cartilage (D) followed by the appearance of joint domains (arrowheads, B) and then tendons domains (arrows, E). G–I, Expression of *Ccn3* first appears in the developing tendons (G,H) and then extends to the developing joints (I).

In view of these results, we selected CCN1, CCN2 and CCN3 for further analysis. The absence of detailed expression studies during digit development, prompted us to analyze their expression during normal digit formation. As show in Fig. 3A, *Ccn1* appeared as a most precocious marker of the digit blastemas. As development progressed, transcripts accumulated in the developing joints and in the tendon blastemas, whereas expression in the digit rays was restricted to the distal phalanx in course of differentiation (Fig. 3B). When all of the phalanxes had formed, expression of *Ccn1* was restricted to the differentiating joints and tendons (Fig. 3C). *Ccn2* was expressed in the prehypertrophic cartilage of the digit rays and, with a lower intensity, in the developing joints (Fig. 3D, 3E), but the distal tip of the digits where prechondrogenic cell condensation occurs lacked identifiable labeling. The expression of *Ccn3* was intense in the developing tendons and in the developing joints, whereas the chondrogenic blastemas were negative fo *Ccn3* (Fig. 3G, 3H, 3I).

**Figure 4:**
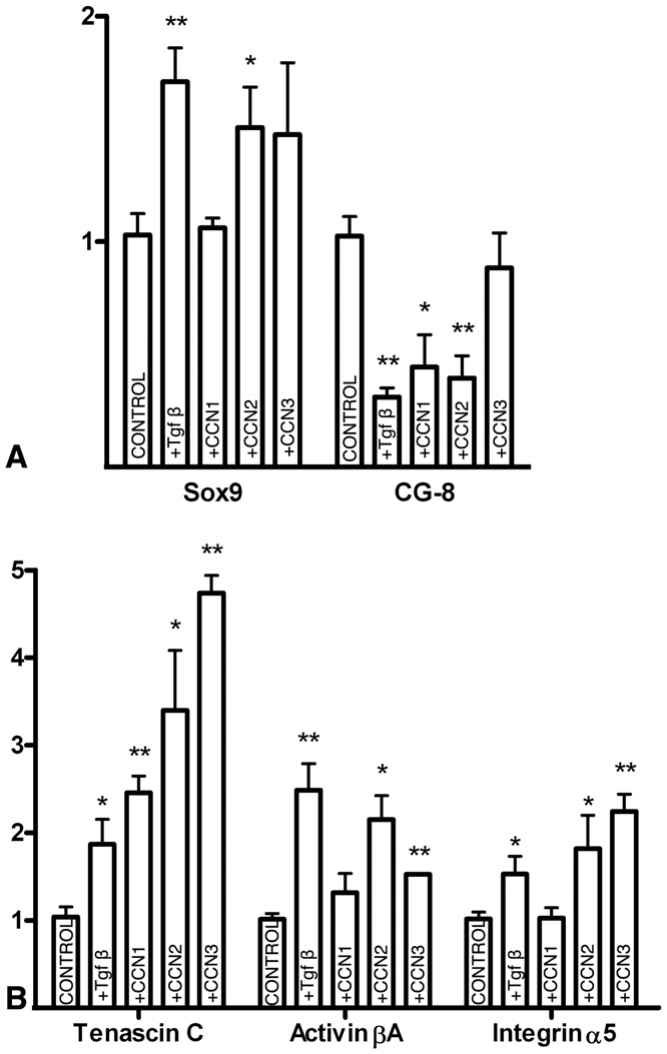
Regulation of chondrogenic factors by CCN proteins. Charts showing the regulation of *Sox9*, *Scleraxis*, and *CG-8* (A); and *Tenascin C*, *Activin βA* and *alpha 5 Integrin* (B) in the interdigital mesoderm 6 hr after the implantation of beads bearing PBS (first column, control), Tgfβ (second column), CCN1 (third column), CCN2 (fourth column) and CCN3 (fifth column). (*) p-value≤0.05; (**) p-value≤0.01.

**Figure 5:**
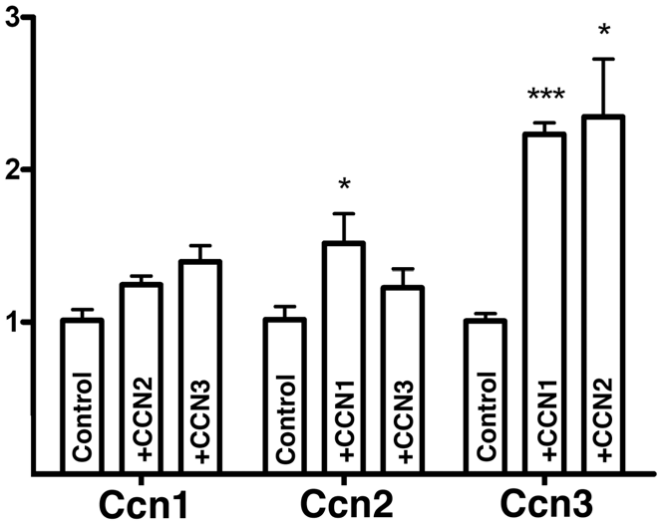
Chart showing the regulation of the *Ccn1*, *Ccn2*, and *Ccn3* genes in the interdigital mesoderm 6 hr after the implantation of beads bearing PBS (control), CCN1, CCN2 or CCN3. (*) p-value≤0.05; (***) p-value≤0.001.

In view of the specific expression domains observed in the developing digits and taking into account that some *Ccn* genes are direct targets of Tgfβ signaling [60], we decided to explore whether interdigital implantation of CNN beads could replicate the effect of Tgfβs in inducing an ectopic digit. We observed that implantation of beads bearing recombinant CNN1, CCN2 or CCN3 or a mixture of the three factors was not followed by the formation of ectopic cartilages (n = 80). However, analysis of gene expression at 6 hr after interdigital implantation of beads bearing CCN1, CCN2 or CCN3 revealed significant changes in the expression of pro-chondrogenic factors (Fig. 4A, 4B). CCN1 beads induced the upregulation of *Tenascin C* and the downregulation of *CG-8*. CCN2 exhibited the same effects as CCN1 but caused an additional moderate upregulation of *Sox9*, *Activin βA* and *alpha 5 Integrin*. CCN3 induced intense upregulation of *Tenascin C* and, at a reduced level, of *alpha 5 Integrin*, S*ox9*, and *Activin βA*. The possible crosstalk between CCN1, CCN2 and CCN3 was also studied (Fig. 5). Consistent with the temporal pattern of expression, both CCN1 and CCN2 induced a two-fold increase in the expression of *Ccn3*. CCN1 also caused a mild (1.5-fold) increase in the expression of *Ccn2*.

### Transforming Growth Factor Signaling Pathways (Table 4)

**Table 4:** Regulation of genes in the Tgf-β signaling pathway at 1, 3, 6, and 10 hr after interdigital implantation of beads bearing Tgf-β1.

		Fold changes vs control			
Gene	GenBank	1 hr.	3 hr.	6 hr.	10 hr.
**TGFβ Signalling**					
***Activin βA***	NM_205396	1.39±0.20	1.59±0.02*	2.49±0.30**	10.83±1.90**
***Activin βB***	NM_205206	1.37±0.45	1.11±0.19	0.80±0.18	0.52±0.06*
*ACVR2B*	NM_204317	1.07±0.04	1.01±0.02	0.90±0.05	1.63±0.48
*ACVLR1 (ALK1)*	BU258674.1	0.89±0.02	2.14±0.32**	1.46±0.73	0.97±0.13
*ACVR1 (ALK2)*	NM_204560	1.04±0.06	1.06±0.10	1.26±0.15	0.98±0.14
*BmpR1A (ALK3)*	NM_205357	0.96±0.03	1.43±0.08	1.05±0.23	1.52±0.11
***TgfβR1*** * (ALK5)*	NM_204246	0.71±0.08	1.30±0.15	1.01±0.03	0.75±0.10
*BmpR1B (ALK6)*	NM_205132	0.73±0.04	0.97±0.14	0.90±0.06	2.01±0.16*
*BAMBI*	XM_425974	0.94±0.01	0.95±0.02	1.15±0.45	0.92±0.08
*Gremlin 1*	NM_204978	0.81±0.14	1.20±0.04	0.79±0.14	0.93±0.18
*Neogenin*	XM_413704	1.04±0.09	1.11±0.11	1.17±0.04	0.86±0.00
***TgfβR2***	NM_205428	0.75±0.10	1.23±0.05	0.83±0.08	0.90±0.20

(*) p-value≤0.05 or (**) p-value≤0.01 or (***) p-value≤0.001 using the expression level of the gene in the contralateral untreated interdigit as the calibrator.

Members of the Transforming Growth Factor superfamily have been identified as chondrogenic triggering signals in vivo [14], [15], [61]. Studies using different experimental approaches have conclusively demonstrated a pivotal function of BMP signaling in chondrogenesis via phosphorylation of regulatory SMADs 1/5/8. Here, we decided to analyze the regulation of the type I receptors because ligands in this pathway show functional redundancy [62], [63]. Upregulation of the *BmpR1b* gene was observed at 10 hr ABI. In contrast, the expression of *BmpR1a* was maintained without modifications throughout the entire period covered in this study. The relatively late upregulation of *BmpR1b* gene observed in our model is remarkable in comparison to the demonstrated function of BMPs to direct differentiation of mesenchymal cells into cartilage [64] and to induce ectopic cartilage in a variety of tissues of adult animals [61]. To unravel this apparent contradiction, we extended our expression study to other type I receptors and BMP regulators. We observed a transient up-regulation of *ALK1* 3 hr after the treatment consistent with the precocious phosphorilation of Smad 1/5/8 proteins previously observed during digit development [65]. This finding supports observations showing that during chondrogenesis, Tgfβ signaling through p-Smad 2 and 3 acts as a potent promoter of BMP signaling [37], [65], [66]. Additionally, these results explain the synergistic effect of combined treatments using Tgfβs and BMPs to induce cartilage differentiation of mesenchymal stem cells [67], [68]. Here, we did not find changes in the expression of the *Neogenin* gene, a component of cell membranes that potentiates the activity of BMP receptors to activate Smad phosphorylation during skeletogenesis [69]. However, as mentioned above, *Gata 5* a transcription factor proposed to promote the positive influence of Tgfβs on BMP signaling-induced chondrogenesis [37], was upregulated as early as the first hour ABI.

Activins and Tgfβs, are members of the Tgfβ superfamily and share a canonical intracellular signaling mechanism via phosphorylation of Smad 2 and 3. We have previously established that activation of Smad 2 and 3 by these cytokines is the first step in our experimental model of ectopic chondrogenesis [15], [65]. Here, we observed that the *βA activin* subunit [15], but not the *βB* subunit or representative receptors of this pathway, such as *Tgfβ receptor I*, *Tgfβ receptor* II or *Activin receptor 2B*, were not regulated in the first 10-hr period ABI.

## Discussion

The induction of interdigital extra-digits, employed in this study, serves as a powerful experimental model system to characterize the molecular cascade accounting for chondrogenesis in vivo. Unlike current in vitro chondrogenic assays (see [70]), in our model, cell differentiation exactly replicates the events occurring under physiological conditions to form a digit. We cannot discard, however, that the interdigital mesenchymal cells, at the studied stages, have reached a certain degree of specification towards chondrogenesis through the patterning signals operating in the limb preceding the studies stages. However, interdigital cells at the studied stages have potential to differentiate not only in cartilage but also in all other connective tissues [71].

Our results emphasize the function of cell motility in the earliest stages of chondrogenesis. The ectopic prechondrogenic aggregate obtained here is formed from a mesoderm population that is fated to cell death [72] in the absence of increased proliferation. We show that activation of the F-actin cytoskeleton and upregulation of *RhoC*, together with upregulation of the first skeletal markers occur preceding condensation. Furthermore, and most importantly treatment with Cytochalasin D inhibits chondrogenesis. Taken together, these findings are consistent with the results of live imaging studies of chondrogenic cultures performed by Barna and Niswander [64], which have shown that clustering by cell migration is the first event to commit the mesoderm to form a prechondrogenic aggregate.

Our findings are consistent with a role of lectins as precocious triggering signals for chondrogenesis, as proposed recently by Bath et al. [34]. According to these authors, the initiation of chondrogenesis in the limb bud is activated by a balance between the prochondrogenic influence of CG1A and the antichondrogenic effect of CG-8. Consistent with this interpretation, we observed a precocious downregulation of CG-8 after Tgf β treatments. At diference of the study of Bath et al., we were not able to detect an upregulation of prochondrogenic lectins at the most precocious stages of chondrogenic differentiation. However, the high basal expression of CG1A in the interdigital mesenchyme could make it unnecessary its upregulation to initiate chondrogenesis.

A additional observation of this study concerns the implication of CCN matricellular proteins in skeletogenesis. CCNs comprise a family of 6 cysteine-rich secreted proteins present in extracellular matrices (reviewed by [73], [74]) that regulate most aspects of cell behavior, including cell adhesion, migration, proliferation, differentiation and survival (review by [75]). According to the results of our study, CCN1, CCN2 and CCN3 emerge as major regulators of digit skeletogenesis, including playing roles in the formation of phalanxes, tendons and joints. These three members share a potent positive influence on the expression of *Tenascin C*. This effect is in agreement with their common expression in developing tendons and joints, which together with the digit aggregates, are zones of high concentrations of Tenascin C [76]. We also observed that the *Ccn1* gene, unlike *Ccn2* and *Ccn3*, is a specific and precocious marker of the digit blastemas. Interestingly, this expression domain correlates not only with a positive influence on *Tenascin C* gene expression, but also with a negative influence on the expression of the galectin *CG-8*. However, despite the negative influence on *CG-8* expression, CCN1 is not able to substitute for Tgfβs or Activin A in the induction of interdigital cartilage.

In previous studies, CCN1, CCN2 and CCN3 have been implicated in axial and appendicular skeletogenesis [21], [77]–[81]. However, mice deficient in these factors do not exhibit digit dysmorphogenesis, and their skeletal phenotypes are normal, except for abnormal ribs in CCN2-deficient mice [82] and overgrowth of the long bones and joint defects in CCN3-deficient mice [81]. These skeletal phenotypes contrast with the intense expression of these three CCN members in the developing joints and tendons, and in digit blastemas in the case of CCN1. The results of our study show that CCN1 and CCN2 exert the same influence on the expression of *CG-8*, and CCN2 and CCN3 have common regulatory effects on the expression of tendon and joint markers, such as *Tenascin C*, and *alpha 5 Integrin*. Additionally, we show that both CCN1 and CCN2 are positive modulators of the expression of *Ccn3*. Collectively, these finding are indicative of functional redundancy among these factors, which explain the relatively reduced skeletal phenotype of mice deficient in one of the factors.

Beyond the developmental interest of individual data obtained in this study, such as those discussed above, the most relevant finding of this study can be deduced from the collective analysis of the temporal sequence of events observed during the induction of an ectopic digit. Our results show that the differentiation of the embryonic limb mesoderm towards cartilage is preceded by three successive commitment stages.

The initiation of cell condensation occurs within a short 3-hr period that we propose to designate the “pre-condensation stage”, in which the most characteristic cartilage transcription factors, including *Sox9*, *Scleraxis*, and *Barx1*, become upregulated. During this period, *Ephrin A5* and *Big-h3* are relevant modulators of cell adhesion exhibiting significantly upregulation levels. Furthermore, the observed increased distribution of actin filaments along with the upregulation of the small GTPase RhoC are indicative of tissue remodeling. In this period, upregulation of the *Ccn1* and *Ccn2* genes was also observed. Downregulation of galectin CG-8, which exerts a negative influence on chondrogenesis [34], is also a remarkable feature of this period. Together, these findings fit with a period characterized by cell rearrangement in which the undifferentiated mesoderm acquires the initial molecular identity characteristics of connective tissue progenitors.

The second period we identified, which can be termed the “condensation stage”, corresponds to the mesenchymal “condensation period” [83] or “compactation period” [64] that has been well recognized in *in vitro* studies [2]. This period is characterized by increased expression of *Tenascin C* accompanied by increased expression of *NCAM*, *alpha 5 Integrin*. During this period, *Activin βA* which is considered one of the most precocious markers of the digit blastemas [15] is intensely upregulated. Remarkably the Tgfβ extracellular regulator *Ltbp1*, is also upregulated at this period. The paired-box transcription factor Pax1, which is of key importance in the formation of the axial skeleton [2], [43], also becomes up-regulated, though *Baps1/Nkx.3* do not. Consistent with the results of a study by Barna and Niswander [64], a further major characteristic of this period is the activation of BMP signaling, caused by upregulation of BMP receptor genes.

In accord with the upregulation of different pro-chondrogenic factors, we also distinguish a third period that we refer to as the “pre-cartilage period”. This period precedes the upregulation of *Aggrecan* and *type II Collagen* genes, which are considered consensus markers for the onset of cartilage differentiation. The genes upregulated in this period include those observed in the preceding stage, in addition to factors such as extracellular matrix components like fibronectin; the metalloproteinase *Tolloid*; the galectin *CG-1B*; and transcription factors such as *CFkh1*, *Hif1a*, and the Tgfβ co-repressor *Tgif 1*. A major characteristic of this period at the transcriptional level, is the intensification of the expression of *Sox9* from the two-fold increase respect untreated interdigits observed at the precondensation stage to up to 14-fold. This upregulation is considerable higher in comparison with that observed for *Scleraxis*. This increased expression may mark the functional change of Sox9 from regulating cell shape and cell dynamics [64] to controlling cartilage matrix gene expression [1], establishing the divergence between cartilage and fibrous connective tissue condensations. It must be taken into account that the developing fibrous connective tissues and cartilage share similar gene expression profiles during the condensation period (review by [8]), and their developmental divergence is thought to be caused by changes in the expression rate between *Sox9* and *Scleraxis* [47], [84], [85].

## Materials and Methods

In this work, we employed Rhode Island chicken embryos from day 5 to day 10 of incubation (id) equivalent to stages 27 to 36 HH.

### Experimental Induction of Ectopic Digits and CCN Treatments

Ectopic digits were induced by local implantation of heparin (Sigma) beads incubated for 1 hr in 2 µgr/ml rh-TGFβ1 (R&D Systems). For this purpose eggs were windowed at 5.5 id and the bead (raging between 80 and 150 µm of diameter) was implanted in the third interdigit of the right leg bud. The contralateral left limb or limbs treated with beads incubated in PBS, were employed as controls. After manipulation the eggs were sealed and further incubated until processing.

To test the importance of actin microfilaments in the establishment of the prechondrogemnic condensations combined treatments with Tgfβ and Cytochalasin D were performed. Cytochalasin D (Calbiochem) was applied by implantation of acrylic beads incubated for 1 hr in 25 µM Cytochalasin D in dimethyl sulfoxide (DMSO) at the same time than the Tgfβ-bead. Controls substituting the Cytochalasin bead by a PBS bead were also performed.

Treatments were also performed with beads incubated in 400 µgr/ml of human recombinant CCN1, CCN2, or CCN3 (all from PeproTech).

### Skeletal Morphology, Immunolabeling, TUNEL Assay, and Lectin Histochemistry

The skeletal morphology of limbs treated with Tgfβ beads was studied in whole mount specimens stained with Alcian green as described previously [14].

The presence of prechondrogenic condensation in the treated interdigits was studied with fluorescein isothiocyanate labeled Peanut agglutinin (PNA) staining. For this purpose the autopods were fixed in cold acetic-alcohol and vibratome section were incubated for 30 min in PNA (Sigma) and washed in PBS. Sections were mounted in 50% glycerol/PBS.

Actin cytoskeleton labeling was performed in vibratome sections of specimens fixed in 4% paraformaldehyde using 1% or Phalloidin-TRITC (Sigma). Some specimens were also immunolabeled with phospho-SMAD 2 or phosmo-SMAD 1/5/8 polyconal antibodies (both from Cell Signaling). In these cases we first performed the corresponding immunolabeling followed by Phalloidin staining.

Changes in cell death after Cytochalasin D treatments was analyzed by TUNEL assay in vibratome sections of parformaldehyde fixed control and experimental limbs.

### In Situ Hybridization

In situ hybridization was performed in whole mount specimens or in 100 µm vibratome sections of autopods fixed in 4% paraformaldehyde. Samples were treated with 10 µg/ml of proteinase K for 20–30 minutes at 20°C. Hybridization with digoxigenin labeled antisense RNA probes was performed at 68°C. Alkaline phosphatase-conjugated anti-digoxigenin antibody (dilution 1∶2000) was used (Roche). Reactions were developed with BCIP/NBT substrate (Roche). Probes for *type II Collagen*, and *Aggrecan* were widely employed in previous studies (see [16]). Probes for *Ccn1*, *Ccn2* and *Ccn3* genes were obtained by PCR. The following primers were employed: Chick CCN1: fwd 5′- gtctgcgatgagagcaagg - 3′ and rev 5′- gagtacagcacctgccatcc - 3′; Chick CCN2: fwd 5′- ccagagcagctgcaagtacc - 3′ and rev 5′- tgcagacaccacagaacttagc - 3′; Chick CCN3: fwd 5′- acaactgcgtgttcgatgg - 3′ and rev 5′- gcggaactcaacttgaatcg - 3′.

### Real Time Quantitative PCR (Q-PCR) for Gene Expression Analysis

In each experiment total RNA was extracted and cleaned from specimens using the RNeasy Mini Kit (Qiagen). RNA samples were quantified using a spectrophotometer (Nanodrop Technologies ND-1000). First-strand cDNA was synthesized by RT-PCR using random hexamers, the M-MulV reverse transcriptase (Fermentas). The cDNA concentration was measured in a spectrophotometer (Nanodrop Technologies ND-1000) and adjusted to 0.5 µg/µl. Q-PCR was performed using the Mx3005P system (Stratagene) with automation attachment. In this work, we have used SYBRGreen (Takara) based QPCR. *Gapdh* and *Rpl13* were chosen as the normalizers in our experiments giving identical results. Mean values for fold changes were calculated for each gene. Expression level was evaluated relative to a calibrator according to the 2-(ΔΔCt) equation [86]. Each value in this work represents the mean ± SEM of at least three independent samples obtained under the same conditions. Samples consisted of 12 interdigital spaces. Data were analyzed using one-way ANOVA followed by Bonferroni tests for post-hoc comparisons, and Student-t-test for gene expression levels in over-expression experiments. Statistical significance was set at p<0.05. All the analyses were done using SPSS for Windows version 15.0. Primers for Q-PCR are included as Primers S1. Statistic analysis was performed when genes were upregulated more than 50% or downregulated at least by 40%.

### Flow Cytometry

Control and treated interdigits were dissected free and dissociated to single-cell level by treatment with 0.25% trypsin and 0.25% collagenase for 3 min each. The cellular suspension was then filtered through a 100 µm membrane. 1 million cells (12 interdigits) were used in each test. For propidium iodide (PI) staining the cells were washed twice in PBS and centrifuged at 405 g, 5 min at 4°C. The samples were then incubated overnight at 4°C with 0.1% sodium citrate, 0.01% TritonX-100 and 0.1 mg/ml PI. Cell suspension was subjected to flow cytometry analysis in a Becton Dickinson FacsCalibur cytometer and analyzed with Cell Quest software.

## Supporting Information

Primers S1 (DOC)
