# Supplementary material for: Defining the Earliest Transcriptional Steps of Chondrogenic Progenitor Specification during the Formation of the Digits in the Embryonic Limb
Source: PLoS One. 2011 Sep 13;6(9):e24546. doi: 10.1371/journal.pone.0024546 (PMC3172225; doi:10.1371/journal.pone.0024546)
Supplement: Primers S1 — (DOC) [file pone.0024546.s001.doc]

| **Gene** | **Forward Primer 5’- 3’** | **Reverse Primer 5’- 3’** |
| --- | --- | --- |
| ***Gapdh*** | **ggtggccatcaatgatcc** | **gttctcagccttgacagtgc** |
| ***N-cadherin*** | **acatggaggacaacctctgc** | **tcttctggctcactgcttcc** |
| ***Cadherin 4*** | **aactggaatgccatctaccg** | **ccttgaccacagttaccatgc** |
| ***Cadherin 11*** | **ctctcctgtaatgccgaagc** | **cacaatgcaagcaagaatgg** |
| ***Cadherin 13*** | **tgcggtataacatcctcaagc** | **tcacgatccagcagtacagg** |
| ***Cadherin 7*** | **cagcatattgcaaggacaacc** | **tggtccttggcttctctatcc** |
| ***ITGA4*** | **ttgcacctggaagctactcc** | **aggctgtcgctcttacttcg** |
| ***ITGAV*** | **atggatcgaggttctgatgg** | **atagcactgccgaatcttgc** |
| ***ITGβ1*** | **acttgccttggtgtctgtgc** | **catgcattcctgagaacacg** |
| ***ITGβ3*** | **gcaccttgtccagagactcc** | **cacgcacctccagttctacc** |
| ***ITGA1*** | **acgctacttcagtgcctaacg** | **catagagcggtccacatgc** |
| ***ITGA5*** | **tggggacaccgacctggatgg** | **cgctggcatggactatggggc** |
| ***ITGβ5*** | **cttcaccacagacgatgtgc** | **gacgcgctgtactcattgg** |
| ***NCAM*** | **gagttcgatgagcctgaagc** | **gagtgccattctccttcacc** |
| **β *Catenin*** | **gcagcagcagtcatatctgg** | **acaggacttgcgttgtgtcc** |
| ***RhoA*** | **cagcaggacaggaagactacg** | **caagatgataggcacgttgg** |
| ***RhoC*** | **caggaagaagctggtgatcg** | **aacacagttggcacgtagacc** |
| ***Ephrin-A2*** | **tcttcaccagcaacaactcg** | **ccagcagagtccagaagacc** |
| ***EPH receptor A4*** | **gctcctgtgtcaacaactcg** | **agcattgcacagacagttgc** |
| ***Ephrin-A5*** | **cggtgccagaagataagacc** | **acttcagcggtccattcg** |
| ***EPH receptor A7*** | **agtcctcctgctgacaatgg** | **ccacaaggaacacattcacc** |
| ***CG-8*** | **tgcaacaccttggagagg** | **ggctaattctgtgattgtagagc** |
| ***CG-1A*** | **gagcaaggactggttgttacc** | **gagtgtgctgctgtccttcc** |
| ***CG-1B*** | **ggataatcgcaccgaatgc** | **cacaatgaggttcacatcacc** |
| ***Sox9*** | **gaggaagtcggtgaagaacg** | **gatgctggaggatgactgc** |
| ***Sox8*** | **gccagaacattgacttcagc** | **attgagcggcaggtactgg** |
| ***Scleraxis*** | **caccaacagcgtcaacacc** | **cgtctcgatcttggacagc** |
| ***CFKH-1*** | **ctacatcgcgctcatcacc** | **ctccatgatgaactggtagatcc** |
| ***PRRX1*** | **aggaagcagagaaggaacagg** | **gttgactctgcgtgcaagg** |
| ***PRRX2*** | **ggtctttgagcggacacatt** | **gtaggacttgaggagcgacg** |
| ***GATA5*** | **cagaagaggctgtcttcatcg** | **ttcggcgttccttctcc** |
| ***GATA6*** | **tgtcctgtgccaactgc** | **gacgtagatgttggagtcatagg** |
| ***NKX3.2 (Bapx1)*** | **ctgacgcctttctccatccaggc** | **cgctctcgggctcctcgctc** |
| ***Runx2*** | **tcttcagctccgtgacacc** | **ctgacctcgctcatcttgc** |
| ***BARX1*** | **gcttcgagaagcagaagtacc** | **tcatgcgcctgttctgg** |
| ***Pax1*** | **gcgactacaagcagggagac** | **atcctgctgatggagctgac** |
| ***Pax9*** | **cgtcggtcagctctatcagc** | **ggctgatgctgcttgtagg** |
| ***SnoN (SKIL)*** | **acctgcctcctatccagagc** | **ccacctcttgcagaatgagc** |
| ***Tgif1*** | **ctctcctaccacgaggatgc** | **gtgcaacatccaccagtagc** |
| ***Hif1α*** | **gacaaggcatccattatgagg** | **cagctccttctccatgttgg** |
| ***Tenascin C*** | **ggagcatacggtgaatgagg** | **agcaggtccttgatgtctgg** |
| ***Versican*** | **ggccttacaaccacaagagg** | **ctgcatgaggaagaatgtgg** |
| ***Decorin*** | **atggctccaacagatgatcc** | **tccagcagagttgtgtcagg** |
| ***Tenomodulin*** | **atgcagaagcatccaagacc** | **aagagcacgaggatgagagc** |
| ***Glypican 3*** | **ggagaagtaccaggcagtgg** | **cagcattctggatgacaagg** |
| ***Fibronectin1*** | **actgtctccaccaaccaacc** | **agcagtggtcactcggtagc** |
| ***Ltbp1*** | **tgagtgctgttgcttgtatgg** | **gaacaggaatgttgcacagc** |
| ***βig-h3*** | **cttctgacctcaacagcttgc** | **gatcctgttcagcatctctcg** |
| ***Tll1*** | **tatgtggagattcgcagtgg** | **aacttcaggcacttccgtacc** |
| ***MT3-MMP (MMP16)*** | **atgaagaagcctcgatgtgg** | **ttgtgttgccacttctgtcc** |
| ***Ccn1*** | **gtctgcgatgagagcaagg** | **cttcacgatggcaatcagc** |
| ***Ccn2*** | **gactactgagtggagtgcttgc** | **gctctgcttctccagtctgc** |
| ***Ccn3*** | **acagaatggagtgcttgttcc** | **gcttcaccatctcacactgc** |
| ***Ccn4*** | **agacctccacaaccatgacc** | **tctgtgacaaggctgactcc** |
| ***Ccn5*** | **tgctctgcaggagtgatacg** | **ggaagcagacggagaagagg** |
| ***Ccn6*** | **ggttgctgtaggatgtgagc** | **tcgcaccactgatacacagg** |
| ***BmpR1B (ALK6)*** | **cctcctacgaggacatgagg** | **gcactccatcatgagcttcc** |
| ***BmpR1A (ALK3)*** | **ttccagtgcaaggattcacc** | **atcaagcggtggtaatgtcg** |
| ***Neogenin*** | **atctccatcaccaggtcagc** | **aacggattctgtggaattgg** |
| ***TgfβR1 (ALK5)*** | **gttattgctggacctgtctgc** | **tcttcttcacttggcacacg** |
| ***TgfβR2*** | **accgcactcacaagaagagg** | **gttgatgttgttggcacagg** |
| ***ACVR2B*** | **ggagcaatcaacttccaacg** | **ccactggaccatcaactgc** |
| ***Activin βA*** | **gctgactgtccatcatgtgc** | **actgcttccaccatctcagg** |
| ***Activin βB*** | **tggatcatagcaccatcagg** | **gcattcggtactgattcacg** |
| ***ACVLR1 (ALK1)*** | **agcgactacctggacattgg** | **tgtaggactcgaagcagtcg** |
| ***ACVR1 (ALK2)*** | **gcattaacgatggtgctaagg** | **acagcttggtcaggagatgg** |
| ***BAMBI*** | **actgcaagatcaacgacagc** | **caccatgcattccaagtcc** |
| ***Gremlin 1*** | **agtcgcaccattatcaacagg** | **ttgcagaaggaacaagactgg** |
